# SOFTWARE-ASSISTED IMAGE ANALYSIS FOR IDENTIFICATION AND QUANTIFICATION OF HEPATIC SINUSOIDAL DILATATION AND CENTRILOBULAR FIBROSIS

**DOI:** 10.1590/0102-672020210002e1608

**Published:** 2021-10-18

**Authors:** Douglas Mesadri GEWEHR, Allan Fernando GIOVANINI, Sofia Inez MUNHOZ, Seigo NAGASHIMA, Andressa de Souza BERTOLDI, Ana Cristina Lira SOBRAL, Fernando Bermudez KUBRUSLY, Luiz Fernando KUBRUSLY

**Affiliations:** 1 Mackenzie Evangelical Faculty of Paraná, Curitiba, Paraná, Brazil;; 2 Denton Cooley Institute of Research, Science and Technology, Curitiba, Paraná, Brazil; 3 Curitiba Heart Institute, Curitiba, Paraná, Brazil; 4 Laboratory of Experimental Pathology of Health and Biological Sciences, Pontifícia Universidade Católica do Paraná, Curitiba, Paraná, Brazil

**Keywords:** Image processing, computer-assisted, Liver fibrosis, Sinusoids, Processamento de imagem, Processamento de imagem assistido por computador, Fibrose hepática, Sinusoides

## Abstract

**Background:**

Heart dysfunction and liver disease often coexist because of systemic disorders. Any cause of right ventricular failure may precipitate hepatic congestion and fibrosis. Digital image technologies have been introduced to pathology diagnosis, allowing an objective quantitative assessment. The quantification of fibrous tissue in liver biopsy sections is extremely important in the classification, diagnosis and grading of chronic liver disease.

**Aim:**

To create a semi-automatic computerized protocol to quantify any amount of centrilobular fibrosis and sinusoidal dilatation in liver Masson’s Trichrome-stained specimen.

**Method:**

Once fibrosis had been established, liver samples were collected, histologically processed, stained with Masson’s trichrome, and whole-slide images were captured with an appropriated digital pathology slide scanner. After, a random selection of the regions of interest (ROI’s) was conducted. The data were subjected to software-assisted image analysis (ImageJ^®^).

**Results:**

The analysis of 250 ROI’s allowed to empirically obtain the best application settings to identify the centrilobular fibrosis (CF) and sinusoidal lumen (SL). After the establishment of the colour threshold application settings, an in-house Macro was recorded to set the measurements (fraction area and total area) and calculate the CF and SL ratios by an automatic batch processing.

**Conclusion:**

Was possible to create a more detailed method that identifies and quantifies the area occupied by fibrous tissue and sinusoidal lumen in Masson’s trichrome-stained livers specimens.

## Resumo

**Racional:**

Tecnologias de imagem digital têm sido introduzidas ao diagnóstico patológico, permitindo avaliações quantitativas objetivas. A quantificação de tecido fibroso em biópsias de fígado é extremamente importante para a classificação, diagnóstico e graduação de doenças crônicas hepáticas.

**Objetivo:**

Criar um protocolo computadorizado semi-automático para quantificação de fibrose centrolobular e dilatação sinusoidal em amostras de fígado coradas por Tricrômico de Masson.

**Método:**

Uma vez instaurada a fibrose, amostras de fígado foram coletadas, processadas histologicamente, coradas por Tricrômico de Masson e WSI (Whole Slide Images) foram capturadas por scanner digital patológico apropriado. Uma seleção aleatória das regiões de interesse (ROI) foi realizada. Os dados foram submetidos a uma análise de imagem assistida por software (ImageJ^®^).

**Resultados:**

A análise de 250 ROIs permitiu obter-se empiricamente as melhores configurações capazes de identificar fibrose centrolobular (FC) e lúmen sinusoidal (LS). Após o estabelecimento das configurações de padrão de cor, uma Macro de autoria própria foi gravada para definir as medidas (área da fração e área total) e calcular as razões de FC e LS por processamento em grupo/lote (batch mode).

**Conclusão:**

Foi possível criar um método detalhado capaz de identificar e quantificar a área ocupada por tecido fibroso e lúmen sinusoidal em espécimes de fígado coradas por Tricrômico de Masson.

## INTRODUCTION

Heart failure is a major health care concern and its prevalence will increase due to an aging society and the advance of medical therapies. Heart dysfunction and liver disease often coexist because of systemic disorders and diseases that affect both organs as well as the presence of complex cardio-hepatic interactions. Type 2 Cardiohepatic Syndrome (CHS) is characterized by chronic impairment of cardiac function leading to chronic liver injury, which is commonly referred to as congestive hepatopathy^24^. Any cause of right ventricular failure may precipitate hepatic congestion, leading to decreased hepatic blood flow and increased hepatic venous pressure. Histologically, passive liver congestion is reflected by sinusoidal dilatation, congestion, hepatocyte atrophy and fibrosis, most prominent in zone 3, also called the centrilobular region^12^.

In recent years, digital image technologies have been introduced to pathology diagnosis, allowing an objective quantitative assessment, more accurate rather than instead of the qualitative or semi-quantitative analysis that have been done in the conventional visual assessment^13^ ^,^ ^19^ ^,^ ^28^.

Numerous software programs are used daily in laboratories to analyze biological images. Unfortunately, these software programs are expensive and do not allow measurement of features beyond those that are already built in^15^ ^,^ ^17^ ^,^ ^19^ ^,^ ^25^. With the emergence of digital scanning, the histopathological samples can then be handled as digital data instead of conventional glass slides. As a result, the software-assisted analysis became more precise, as it contained the whole slide imaging in a digital file and allows the pre-processing of the regions of interest (ROI), and more advisable, since there is the possibility of using several software’s to analyses the ROI’s obtained from the whole slide imaging^13^.

Few studies involving the quantitative measurement of software-assisted liver fibrosis have been published^4^ ^,^ ^6^ ^,^ ^9^ ^,^ ^15^ ^,^ ^16^ ^,^ ^18^ ^-^ ^22^ ^,^ ^25^. Even scarcer are studies evaluating the centrilobular fibrosis and, to the best of our knowledge, no standardized sinusoidal lumen area quantification method has been described with Masson’s trichrome-stained livers specimens^13^.

In this paper, software-assisted image analysis was applied to the quantitative assessment in an experimental rat model of passive liver congestion induced by right ventricular hypertrophy. Here, we propose a semi-automatic computerized protocol to quantify any amount of centrilobular fibrosis and sinusoidal dilatation in liver Masson’s Trichrome-stained specimens.

## METHODS

The study protocol was approved by the Ethics Committee on the Use of Animals, Mackenzie Evangelical College of Paraná (Faculdade Evangélica Mackenzie do Paraná - FEMPAR). All experimental protocols were performed in compliance with the National Institutes of Health (NIH) guidelines for the care and use of laboratory animals (NIH Publication no. 85723, revised 1996) and conform to the previously described principles and regulations for animal experimentation of Experimental Physiology (Grundy, 2015), and all steps were taken to minimize the animal’s pain and suffering during the experiments. Institutional ethical approval code: 2577/2020.

### Liver samples

Fifty male Wistar rat livers (*Rattus norvegicus*) were used for the development of this new analysis methodology. These animals were submitted to monocrotaline inoculation for the induction of pulmonary arterial hypertension, right heart hypertrophy and, consequently, congestive hepatopathy, previously reproduced by our research group, using the same protocol^10^.

### Histological preparation

The livers were fixed in a 10% buffered formalin solution for 48 h. The left and right lateral hepatic lobes were trimmed in cross section. After fixation, the tissue was embedded in paraffin blocks, and later two 4 *µm* coronal histological sections were obtained for each animal. The histological sections were stained with Masson’s Trichrome (MT) and Hematoxylin-Eosin (HE) and mounted on glass slides. One slide of liver sample was confectioned per animal, totalizing 50 slides for analysis.

### Digital slide scanner and image acquisition

Whole-slide images of liver tissue were obtained with a digital pathology scanner (Axio Scan Z1, Zeiss, Jena, Germany, 40X) and the images were analyzed using the ZEN 2.3 (blue edition) software (Carl Zeiss Microscopy GmbH^©^, 2011), which allows the user navigate through the Carl Zeiss Image and perform geometric and quantitative measurements.

After scanning the MT liver sections with the Axio Scanner, 5 histology regions of interest (ROIs) per slide (images taken at full resolution with a ROI set at 1364x1364 pixels, pixel size is 4.55 µm x 4.55 µm), with the centrilobular vein in the center of each ROI, were obtained randomly from across the entire digital slide as described above, totalizing 250 ROIs. These images were used to quantify centrilobular fibrosis and sinusoidal area.

We have made a series of tests on images from 250 ROI’s. All of them were assessed by different investigators. The images were subsequently analyzed using in-house macros in batch mode of ImageJ^®^ (version 1.53e, ^©^National Institutes of Health, USA).

### Congestive hepatic fibrosis score assessment

The histopathologic evaluation was performed by two different pathologists using the Congestive Hepatic Fibrosis Score (CHFS) system^7^. It is a semi-quantitative pathological classification method. Based on the pattern of fibrosis, scores of 0, 1, 2, 3, and 4 may be assigned as follows: score 0, no fibrosis; score 1, central zone fibrosis; score 2, centrilobular and portal fibrosis; score 3, bridging fibrosis; score 4, cirrhosis.

### Statistical analysis

The CHFS were expressed in median±interquartile range. The Kolmogorov-Smirnov test was used to assess whether the variables were normally distributed. ANOVA one-way followed by Tukey test was used to intergroup comparison since the data were normally distributed. The statistics were carried out using Action Stat^®^ software (version 3.7, Estatcamp Team (2014), Software Action, Estatcamp - Statistics and Quality Consultancy, São Carlos - SP, Brazil) and the graphics were made using MedCalc^®^ (version 19.3.1, MedCalc Software Ltd^©^, 1993-2020).

## RESULTS

**FIGURE 1:**
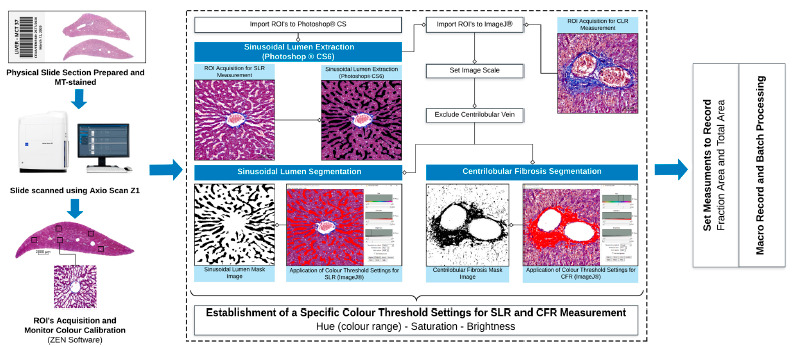
Flow diagram showing methodology of software-assisted image analysis for quantification of centrilobular fibrosis and sinusoidal lumen ratio. The methodology starts from the glass slide stained by Masson's Trichrome, followed by digitization by the Azio Scan Z1 equipment; ROI acquisition and monitor calibration (Zen Software); destination of the image to Photoshop CS6; LS image extraction and segmentation; importing the images into the ImageJ® program; determination of image scale; exclusion of the centrilobular vein image; establishing a specific color standard for RFC and RLS; finalization by recording the measurements and processing by the batch function.

All images were analyzed following the algorithm illustrated in Figure 1, using an in-house developed macroinstruction (Macro), recorded and developed to quantify absolute value in micrometers and the percentage of centrilobular fibrosis area and sinusoidal lumen area compared to the total amount of tissue area within a ROI, allowing the measurement of the CFR and SLR in each visual field. The means of five visual fields were compared.

CFR=centrilobular fibrosis ratio; SLR=sinusoidal lumen ratio; ROI=regions of interest

The ROI’s had some variations in color properties, which occur due some differences in the histological processing of the tissue sample and may also occur during digital image acquisition (camera settings). Because of that, the monitor’s colours were previously calibrated using ZEN 2.3 (blue edition) software.

### Common steps in image processing

For all images to be analyzed, the macro first sets a scale for the image (4.55 µm/pixel). Then, the centrilobular vein was extracted manually and filed with white pixels. After obtaining all the individual components and correcting the images of the areas to be excluded, the macro applies a specific color threshold algorithm and assesses the areas of all components in µm^2^ and percentage. The threshold used by the macro was set empirically by analyzing a test set of 250 ROI’s and selecting the threshold which identifies the components best, minimizing the interference of color variations between slides.

### Sinusoidal lumen segmentation

To quantitatively measure sinusoidal lumen area, it is necessary to segment sinusoids from the trabecula structure. However, as cytoplasm texture depends on the conditions of the cells (i.e. steatosis, atrophy, fatty metamorphosis, dysplasia) and specimens (staining time or specimen fixation), the automatic extraction could be a difficult task, if you are searching for a good accuracy.

**FIGURE 2:**
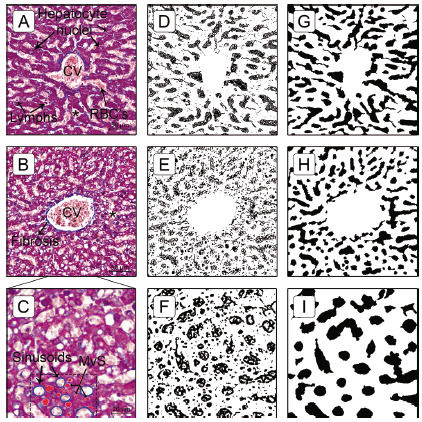
Centrilobular ROI images and respective masks for sinusoidal lumen segmentation: A, B and C) MT-stained specimen of liver tissue; D, E and F) sinusoidal mask’s (binary image) obtained after color threshold and smoothing application using ImageJ^®^, without previously sinusoidal lumen extraction; G, H and I) sinusoidal mask’s (binary image) obtained color threshold and smoothing application, with a previously sinusoidal lumen extraction using Adobe^®^ Photoshop^®^; white pixels indicate the trabecula and centrilobular vein, and the black pixels the sinusoids; G) typical appearance of fat droplets, filled with red pixels, and sinusoids, outlined in blue pixels, inside the dashed rectangle, in a high magnification.

To solve the task of measurement of sinusoidal dilatation, we developed an accurate and simple method composed of two main steps: sinusoidal lumen extraction and sinusoidal feature measurement by a specific color threshold algorithm. The sinusoidal lumens were manually extracted using the Quick Selection Tool (size 10 px; hardness 50%; spacing 50%) in Adobe^®^ Photoshop^®^ CS6 (version 13.0, © Adobe Systems Incorporated, 1990 - 2012) and filled with black pixels. The black pixels indicate the sinusoidal lumen and the white pixels the trabecular structures and central vein. This step is very important since it eliminates morphological structures impairing the analysis (Figure 2D-F), such as red blood cells, fatty droplets, ballooned hepatocytes and others. The fatty droplets (microvesicular steatosis) interference was eliminated when performing sinusoidal lumen extraction (Figure 2G-I) before ImageJ analysis^®^. Basically, we have successfully eliminated the main interferences associated with the isolation and segmentation of sinusoidal lumen to previous extract using Adobe^®^ Photoshop^®^.

MT=Masson’s trichrome; CV=centrilobular vein; lymphs=lymphocytes; MvS=microvesicular steatosis; RBC’s=red blood cells; *=sinusoids

The second step was to apply a threshold algorithm to identify and measure the area occupied by black pixels. The threshold application settings in ImageJ^®^ software for measuring sinusoidal lumen area (black pixels) were: hue 0-255, saturation 0-255, brightness 0-50.

### Centrilobular fibrosis segmentation

A MT-stained image of liver tissue specimen is shown in Figures 2 A, B and C. The connective tissue/fibrosis is stained blue, hepatocyte nuclei (also lymphocytes and Kupffer cells) are stained dark red/purple and cytoplasm is stained red/pink/purple. Sinusoids and fat droplets generally appear as white areas. A trabecula was considered as a series of cells segmented by sinusoids and stromata. Therefore, because the principle of the histological preparation technique, the quantification of liver fibrosis, in this case specifically, centrilobular fibrosis, depends on color discrimination between blue (collagens fibers) and red/purple (parenchyma).

The developed method for quantitatively measuring the fibrosis area consisted in one main step, applying a specific color threshold, after setting the image scale and extracting the centrilobular vein. Color threshold was carried out visually using the default setting histogram and manually adjusted to incorporate the maximum degree blue-stained structures, checking which one appears to identify them best. We applied a smoothing step to the representative ROI’s to reduce noise, improving the blue-stained structures visualization and to define more accurately the color threshold settings. After analyzing 250 ROI’s we empirically obtained the best applications settings to identify the centrilobular fibrosis. The application settings obtained for measuring fibrosis were: hue 140-190, saturation 0-255, brightness 0-248.

**FIGURE 3:**
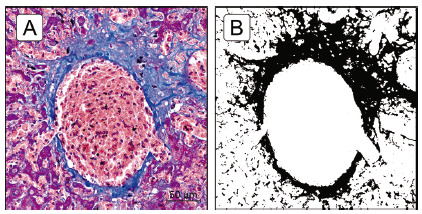
Scattered centrilobular fibrosis: A) representative MT-stained section; B) fibrotic zone mask image using ImageJ^®^ and the black pixels indicate fibrotic zones.

**FIGURE 4:**
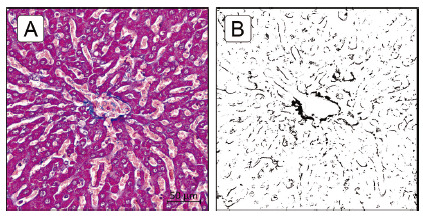
Perisinusoidal fibrosis: A) representative Masson’s trichrome-stained section; B) after image processing and analysis, though the fibrotic zone mask, it is possible to identify the perisinusoidal and the fine intercellular fibrous tissue ramification

The present method was able to identify and quantify, thought the contrast between blue and red/purple-stained structures (collagen fibers and parenchyma, respectively), without affecting each other, the scattered areas of centrilobular fibrosis (Figure 3) as well as the delicate perisinusoidal fibrosis (Figure 4). The color threshold application settings were sufficiently capable on selecting a given range hue (color range), brightness and saturation, without overlapping with other color components.

### Macro record and batch processing - SLR and CFR quantification

After the establishment of the color threshold application settings, an in-house Macro was recorded to set the measurements (fraction area and total area) and calculate the sinusoidal lumen ratio (SLR) and centrilobular fibrosis ratio (CFR) by an automatic batch processing. Once the Macro is loaded onto the ImageJ software and executed within all images (batch mode processing). First, the macro sets a scale to the image. Next, the macros detect the centrilobular vein to be excluded. Then the algorithm determines the total area of the field and the area corresponded to the sinusoidal lumen and centrilobular fibrosis. Finally, the macros calculate the ratio between these measurements by the formulas of SLR and CFR shown below. The mean of the five measures (of the five ROI’s evaluated per slide) was calculated, for each variable, to obtain the CFR and SLR.

The macro is able to analyze multiple images. The batch mode opens the imagens stored in a specific named folder, processes them as described above and writes the results for each image on a separated line, which could be exported to an excel file.

### Congestive hepatic fibrosis score and its correlation with CFR

Histological review of liver samples was performed to clarify the localization of collagen fiber deposition and assess the CHFS. The absence of central zone fibrosis (CHFS 0) was observed in 23 rats (46%). Centrilobular fibrosis, without the portal involvement (CHFS 1), was present in 16 rats (32%), while the concomitant portal fibrosis (CHFS 2) affected the 11 remaining animals (22%).

**FIGURE 5:**
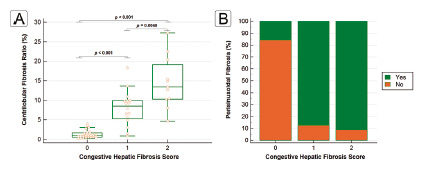
Distribution of the centrilobular fibrosis ratio as a function of the congestive hepatic fibrosis score (A) and frequency of perisinusoidal fibrosis in each CHFS group (B).

The CFR were significantly increased in the CHFS 1 and 2 groups compared to CHFS 0 (1.32±1.07, 8.00±4.45 and 14.55±6.62; p<0.001) and in the CHFS 2 group compared to CHFS 1 (p=0.48). The distribution of the CFR as a function of the CHFS is shown in Figure 5A.

After the software ROI’s processing, the masks obtained (binary images) allowed to characterize more precisely the perisinusoidal fibrosis, present even in CHFS 0. The frequency of perisinusoidal fibrosis in each group of CHFS is summarized in Figure 5B.

## DISCUSSION

Centrilobular fibrosis and sinusoidal dilatation are frequent histological findings seen in hepatic venous outflow obstruction syndromes, such as congestive hepatopathy and veno-occlusive disease^2^. These lesions do not appear only in diseases that result in a liver blood outflow impairment, despite being very common in them, but also in some chronic liver disease. The histological pattern and the severity of centrilobular fibrosis and sinusoidal dilatation varies according to the etiology and natural evolution of the disease and its extent is an important diagnostic and prognostic parameter^26^.

Hepatic fibrosis plays the most important role in the evolutionary process to cirrhosis, independent of the primary etiology. Even with advances in radiological imaging and the development of several putative serum and urinary markers of liver fibrosis, it’s evaluation in liver biopsy specimens remains the ‘gold standard’ for diagnosis, degree assessment and prognosis of liver diseases^1^. Sinusoidal dilatation, a more specific and frequent finding in venous outflow obstruction syndromes, is also very relevant for the evaluation of this condition^2^ ^,^ ^12^.

Semi-quantitative scoring systems have been used in most studies that have relied upon liver biopsy to evaluate changes in fibrosis^5^ ^,^ ^8^ ^,^ ^11^. Comparative characteristics of the different scoring systems is presented by Brunt and Goodmanthe biopsy is often used to assess the severity of the disease in terms of both grade and stage. The stage in most chronic liver diseases relates to the degree of scarring with the end stage being cirrhosis with its clinical complications. The grade relates to the severity of the underlying disease process, with features that vary with the pathogenetic mechanisms. Chronic viral hepatitis has been the object of the most extensive efforts at grading and staging, stimulated by the advent of new forms of therapy. Systems have also been developed for fatty liver disease, allograft rejection and chronic cholestatic diseases, but these have not been as widely used. Simple grading and staging systems for chronic hepatitis, including the IASL, Batts-Ludwig, and Metavir systems, are most appropriate for management of individual patients, while more complex systems such as the Histology Activity Index (HAI)^11^. Recently, Dai et al.^7^ established a simplified histologic scoring system specifically for congestive hepatic fibrosis graduation.

In spite of the efforts made in continuous modifications, citation and revision of these scoring systems to improve diagnosis and classification ability, serious flaws remain. One such problem is that this scoring system is not very precise in fibrosis evaluation as they are subjectively dependent on the visual interpretation of the observer, who must be an experienced pathologist. In clinical practice, pathologists frequently encounter severe problems of large inter- and intra-observer bias^6^ ^,^ ^15^ ^,^ ^22^. 

Therefore, it is clearly fundamental to have a reproducible, objective and rapid method for precisely quantifying the degree of hepatic fibrosis and sinusoidal dilatation in the different compartments of the liver structure. The degree of liver fibrosis is not a ‘single disease state’. The semi-quantitative scoring systems usually are not sensitive enough to appreciate small changes in fibrosis, such as Disse space fibrosis (perisinusoidal fibrosis)^6^ ^,^ ^9^ ^,^ ^27^. Furthermore, even when final fibrotic stage is reached the progression of liver fibrosis and parenchymal remodeling continues^9^.

The development of new morphometric techniques software-assisted to overcome the pitfalls of the conventional histopathological semi-quantitative scoring system have been accomplished, particularly related to liver fibrosis^4^ ^,^ ^6^ ^,^ ^9^ ^,^ ^15^ ^,^ ^16^ ^,^ ^18^ ^-^ ^22^ ^,^ ^25^.

Concerning the sinusoidal dilatation, to the best of our knowledge, no study has been published describing a detailed morphometric method to assess its degree (%) in MT-stained specimens. Ishikawa et al.^13^ proposed a technique to segment, extract and quantify the sinusoids in H&E-stained specimens using an orientation-selective filter with a complex computational algorithm.

The present study aimed to describe a new method for the identification and quantification of delicate perisinusoidal fibrosis as well the scattered areas of fibrosis which are difficult to quantify with the available semi-quantitative scoring system. We primarily focused on assessing zone 3 liver fibrosis, in a model of congestive hepatopathy, although the same protocol can be expanded to assess the fibrosis of other liver zones and compartments.

The core principle of this method is to stain the liver sections with a connective tissue-specific stain, in this case MT, to digitalize the slides and to select aleatory ROI’s. After that, the data were subjected to computational image analysis. 

The strengths of this study include: 1) description of a new technique for measure the sinusoidal lumen area; 2) do not work with grayscale or RGB-threshold image, which could discard much color information, valuable in the assessment of delicate perisinusoidal and scattered fibrosis, without overlapping with other color components; 3) semi-automated nature of the computational image analysis, with a predefined threshold, using a batch mode in ImageJ^®^, able to analyses multiple images at same time. 

The three main limitations of the current study are: 1) the small number of livers specimens to establish a reliable statistical correlation between quantitative and qualitative assessment; 2) not be fully automated, requiring a qualified technician to manually extraction of the sinusoidal lumen, being subjectively dependent on the visual interpretation of the observer; and 3) do not use sirius red stain, the ideal one to bind to collagen proteins^1^. Besides that, this method has two disadvantages: 1) limited information, image analysis provides a quantitative approach to liver fibrosis and cannot replace qualitative determination; 2) cost, image analysis is expensive, since there is a need to scan all slides^23^.

As future perspectives, the aim is to make the sinusoidal segmentation process fully automated, through the implementation of color deconvolution and threshold steps and its cut-off values for the graphic segmentation of these structures, and to compare the effectiveness of this method with manual extraction. 

In fact, the quantification of liver fibrosis as a complement to semi-quantitative indexes of fibrosis, which assesses the distribution pattern of fibrosis, is important for best diagnosis of lesion stage, the most accurate prognosis, and the correct evaluation of different therapies. When sample specimens are adequate, the morphological method, as described, gives accurate fibrosis quantifications through continuous parameters, thus allowing a precise analysis of the variation of liver fibrosis and accurate prognosis of the functional evolution of the liver of in vivo experimental models and in the clinical follow-up patients.

The present paper illustrates, describes and discusses a novel method of quantifying centrilobular fibrosis and sinusoidal dilatation in an experimental model of congestive hepatopathy, based on software-assisted image analysis (ImageJ^®^ application). It provided a reproducible semi-automatic computerized method that precisely identifies and quantifies the area occupied by fibrous tissue in Masson’s trichrome-stained livers specimens. This technique could also be applied in the quantification of fibrosis of other liver zones and compartments. Therefore, to the best of our knowledge, no standardized sinusoidal lumen area quantification method has been described with Masson’s trichrome-stained livers specimens. Our method represents an essential complement to semi-quantitative indexes of fibrosis for better defining the evolution stage of different hepatic diseases and might be of great value to allow the reaching of valid conclusions regarding the applicability of therapies of any etiology for treating livers fibrosis in experimental studies. Thus, it also opens the way for further investigations aimed at extending the use of this technique to other tissue assays.

## CONCLUSION

Was possible to create a semi-automatic computerized method that identifies and quantifies the area occupied by fibrous tissue and sinusoidal lumen in Masson’s trichrome-stained livers specimens. The present method was able to identify and quantify, thought the contrast between blue and red/purple-stained structures, the scattered areas of centrilobular fibrosis and the delicate perisinusoidal fibrosis.
